# Effects of Personal Exposures to Micro- and Nano-Particulate Matter, Black Carbon, Particle-Bound Polycyclic Aromatic Hydrocarbons, and Carbon Monoxide on Heart Rate Variability in a Panel of Healthy Older Subjects

**DOI:** 10.3390/ijerph16234672

**Published:** 2019-11-23

**Authors:** Chin-Sheng Tang, Kai-Jen Chuang, Ta-Yuan Chang, Hsiao-Chi Chuang, Li-Hsin Chen, Shih-Chun Candice Lung, Li-Te Chang

**Affiliations:** 1 Department of Public Health, College of Medicine, Fu Jen Catholic University, New Taipei City 24205, Taiwan; 2 School of Public Health, College of Public Health, Taipei Medical University, Taipei 11031, Taiwan; 3 Department of Public Health, School of Medicine, College of Medicine, Taipei Medical University, Taipei 11031, Taiwan; 4 Department of Occupational Safety and Health, College of Public Health, China Medical University, Taichung 40402, Taiwan; 5 School of Respiratory Therapy, College of Medicine, Taipei Medical University, Taipei 11031, Taiwan; 6 Cell Physiology and Molecular Image Research Center, Wan Fang Hospital, Taipei Medical University, Taipei 11696, Taiwan; 7 Division of Pulmonary Medicine, Department of Internal Medicine, Shuang Ho Hospital, Taipei Medical University, New Taipei City 23561, Taiwan; 8 Research Center for Environmental Changes, Academia Sinica, Taipei 11529, Taiwan; 9 Department of Environmental Engineering and Science, Feng Chia University, Taichung 40724, Taiwan

**Keywords:** heart rate variability, particulate matter, particle-bound polycyclic aromatic hydrocarbons, black carbon, carbon monoxide, healthy older adults

## Abstract

As a non-invasive method, heart rate variability (HRV) has been widely used to study cardiovascular autonomous control. Environmental epidemiological studies indicated that the increase in an average concentration of particulate matter (PM) would result in a decrease in HRV, which was related to the increase of cardiovascular mortality in patients with myocardial infarction and the general population. With rapid economic and social development in Asia, how air pollutants, such as PM of different sizes and their components, affect the cardiovascular health of older people, still need to be further explored. The current study includes a 72 h personal exposure monitoring of seven healthy older people who lived in the Taipei metropolitan area. Mobile equipment, a portable electrocardiogram recorder, and the generalized additive mixed model (GAMM) were adopted to evaluate how HRV indices were affected by size-fractionated PM, particle-bound polycyclic aromatic hydrocarbons (*p*-PAHs), black carbon (BC), and carbon monoxide (CO). Other related confounding factors, such as age, sex, body mass index (BMI), temperature, relative humidity (RH), time, and monitoring week were controlled by fixed effects of the GAMM. Statistical analyses of multi-pollutant models showed that PM_2.5–10_, PM_1_, and nanoparticle (NP) could cause heart rate (HR), time-domain indices, and frequency-domain indices to rise; PM_1–2.5_ and BC would cause the frequency-domain index to rise; *p*-PAHs would cause HR to rise, and CO would cause time-domain index and frequency-domain index to decline. In addition, the moving average time all fell after one hour and might appear at 8 h in HRVs’ largest percentage change caused by each pollutant, results of which suggested that size-fractionated PM, *p*-PAHs, BC, and CO exposures have delayed effects on HRVs. In conclusion, the results of the study showed that the increase in personal pollutant exposure would affect cardiac autonomic control function of healthy older residents in metropolitan areas, and the susceptibility of cardiovascular effects was higher than that of healthy young people. Since the small sample size would limit the generalizability of this study, more studies with larger scale are warranted to better understand the HRV effects of simultaneous PM and other pollution exposures for subpopulation groups.

## 1. Introduction

Over the past decades, cardiovascular disease (CVD), cerebrovascular disease, and cancer have become the top causes of death in Taiwan, in the meantime, the rising trend of cardiovascular disease morbidity and mortality have gradually been the focus of public health policy and epidemiological research in Asian countries [1,2]. Environmental cardiology has been an important field for public health practitioners and clinical researchers to explore the environmental risk factors of CVD and provide useful medical knowledge. However, the cause of CVD is very complex as it could be connected with genetic characteristics and environmental factors. After reviewing over 400 Asian epidemiological studies published in scientific journals in English to explore the health impact of outdoor air pollutants since 1980, Su et al. found that over half of these studies focused on the impact of air pollutants on respiratory diseases, and only about 50 studies assessed the short-term air pollutant exposure on the cardiovascular system [3]. Most Asian cardiologists did not have sufficient knowledge and information about the relationship between CVD and air pollutants compared with their counterparts in the United States and Europe [3,4].

As a non-invasive method, heart rate variability (HRV) has been widely used to study cardiovascular autonomous control. HRV indices can be analyzed in the time-domain and frequency-domain and were proven as important indicators for cardiovascular disease morbidity and mortality. Among them, power in the high-frequency range (HF) mainly reflects the parasympathetic nervous system (PNS), while power in the low-frequency range (LF) indicates the effects of the sympathetic nervous system (SNS) and PNS; and LF/HF is the indicator of global sympathetic and vagal regulation. If SNS and PNS are unbalanced, it will affect the heart contraction that could lead to cardiovascular symptoms [5,6].

Several epidemiological and animal experimental studies have found that exposure to PM_2.5_ (particulate matter with an aerodynamic diameter less than 2.5 μm) could increase the HRV frequency domain indices for the healthy subjects [4,7,8]. Some research also found that occupational polycyclic aromatic hydrocarbons (PAHs) exposure were positively correlated with HRV changes. When workers were exposed to PAHs, one standard deviation (SD) increase in the concentration of 1-hydroxypyrene would bring a 13.6% drop in the 5-min time-domain index SDNN (standard deviation of normal to normal (NN) intervals) [9]. In addition, carbon black (CB) has been found to have an impact on HRV in animal testing. When the mice were given ultrafine CB via trachea, both of SDNN and r-MSSD (the square root of the mean of the sum of the squares of differences between adjacent NN intervals) showed significant decreases with CB exposure of 0.6 mg/kg; mild pulmonary inflammation and myocardial injury were also observed in the group exposed to CB. It was concluded that CB might interfere with the function of the cardiac autonomic nervous system (ANS) in mice and weaken the regulation of the PNS through independent mechanisms [10].

On the other hand, as a recognized cardiovascular toxin, adverse effects of carbon monoxide (CO) range from angina pectoris at moderate exposure concentrations to myocardial infarction at high exposure concentrations [11,12]. When the concentration of carboxyhemoglobin (COHb) was 3% to 6%, adverse health effects were observed in individuals with coronary artery disease (CAD), and the ability of individuals with recurrent exercise-induced angina pectoris (chest pain) to exercise might be impaired. The rise of COHb concentration to 6% or higher also increased the number of CAD patients and the complexity of chronic arrhythmia [13]. Additionally, Tarkiainen et al. found an increase in r-MSSD in relation to acute CO exposure among subjects with stable coronary artery disease [14].

Although previous studies have pointed out that the effect of particle exposure on HRV changes could vary with particle sizes, research on how exposures to particles and its components affect cardiovascular function was limited in Asia at present [15,16,17]. Therefore, this panel study aimed to explore how the personal exposures to micro- and nano-particulate matter, particle-bound PAHs (*p*-PAHs), black carbon (BC), and CO would affect HRV in healthy older adults.

## 2. Materials and Methods

### 2.1. Study Design

The purpose of this study was to assess the changes in HRV caused by particulate and gaseous pollutant exposures among healthy older adults in their living microenvironments. Announcements were posted on campus to recruit volunteers. Seven healthy older people who lived in the Taipei metropolitan area voluntarily participated in the study. The participants were over 55 years old who had not been diagnosed with cardiopulmonary disease or diabetes by physicians, and they did not smoke/drink, as well as having no problems with mobility. Before the study, the research staff would explain the study contents and inform the subjects of matters needing attention. After obtaining the consent of the volunteers, they were asked to sign the consent form before sampling. This study was approved by the ethics committee of Fu Jen Catholic University (approval number C10030).

The monitoring seasons included summer, which was between August and September 2012 and winter, which was between December 2012 and January 2013. The monitoring time of each subject in each season was 3 consecutive days (144 h for 2 seasons). During the monitoring period, the subjects were assisted by the research staff in wearing, self-loading, and unloading an electrocardiogram (ECG) recorder. When the subjects took a bath or went to sleep, they could remove and self-load the ECG recorder by themselves or be assisted by the research staff. In addition, the research staff would put various portable air-pollution monitoring equipment on a 90 cm high table in the subjects’ houses, where they spent most of their time at home. When the subjects were out, they were accompanied by the research staff carrying the portable air-pollution monitoring equipment to measure personal air pollution, temperature, and relative humidity (RH) exposures. The research staff also assisted the subjects in filling in the activity log every day, which included the main location, nature of the activity, indoor ventilation status, and air quality perception. The diary interval of the activity log was 30 min, and the research staff would confirm it again to ensure the information was correct after the completion.

### 2.2. HRV Indices, Air Pollution, and Meteorological Monitoring

The study used a non-invasive portable ECG recorder and analyzer (model E3-8010, MSI, Taiwan) to monitor HRV at a sampling rate of 250 Hz. The time-domain parameters of HRV included r-MSSD, SDNN, and the percentage of adjacent NN intervals that differed from each other by more than 50 ms (pNN50). The frequency-domain parameters included LF, HF, total power (TP), and LF/HF. Together with heart rate (HR), all 8 parameters were calculated in 5 min segments. A trained engineer performed all analyses, and all normal and abnormal findings were checked on the basis of standard criteria to ensure quality control. Regions of noise and artifact were eliminated.

For personal air pollutant monitoring, this study employed a portable aerosol monitor (model 1.109, Grimm, Ainring, Germany) to measure PM exposures in micron-sized ranges. The equipment used light scattering effects to distinguish the particle size and quantity, and the count value was converted into mass concentration through the calculation of dust mass distribution. The study also used a miniature diffusion size classifier (DiSCmini, Matter Aerosol, Switzerland) to assess nanoparticle (NP) (20–700 nm) exposure with a detectable range of 2000 to 500,000 pt/cm^3^. A micro-aethalometer (model AE51, microAeth, San Francisco, CA, USA) was adopted to measure BC exposure (detection range: 0 to 1 mg/m^3^). A photoelectric aerosol sensor (PAS2000CE, EcoChem Analytics, League City, TX, USA) was used to measure *p*-PAHs exposure, where the detection range of the instrument was 0–4000 ng/m^3^. CO exposure was assessed by an electrochemical CO monitor with a detectable range of 0 to 500 ppm (Q-TRAK model 7575, TSI, Shoreview, MN, USA), and a heat stress monitor (QUESTemp 36, QUEST, Louisville, KY, USA) was used to take temperature and RH measurements. While carrying, the complete measurement system weighed approximately 6.6 kg. None of the participants reported time periods when the measurement devices were not with them. All of the pollutant monitoring data were selected to output data in 1 min. In addition to the routine calibration and maintenance of the instruments used in this study, the research staff also performed essential calibration for the instrument readings and flows before and after each monitoring.

### 2.3. Statistical Analysis

The personal air-pollution and HRV monitoring data were documented using Excel, and descriptive statistical analyses were performed with SPSS 18.0 (IBM, Armonk, NY, USA). Data were further processed based on the HRV monitoring time of each subject, and PM_1_ (particulate matter with an aerodynamic diameter less than 1 μm), PM_2.5_, PM_10_ (particulate matter with an aerodynamic diameter less than 10 μm), NP, BC, *p*-PAHs, CO, temperature, and RH values were then obtained simultaneously. In this study, the mass concentration of PM_2.5–10_ was the difference between the concentrations of PM_10_ and PM_2.5_. A similar approach was applied to derive the data for PM_1–2.5_ concentrations. In order to increase the normality and stability of HRV parameters, the natural logarithmic transformation (ln) was performed for HR, r-MSSD, SDNN, pNN50, LF, HF, TP, and LF/HF. In addition, since a part of pNN50 values were 0, the researchers needed to add 1 to all pNN50 values before the natural logarithmic transformation was performed. The addition of 1 could help the natural logarithmic transformation of pNN50 to perform smoothly [18]. In the study, statistical significance was inferred at a *p*-value of 0.05.

The literature review found that most environmental epidemiological studies used a linear model to assess the effects of air pollutants on human health. Since the participants of this research were all independent individuals with repeated measurements over time, and the confounding factors of the monitoring time, temperature, and humidity were nonlinear to the HRV, it was not appropriate to analyze the results with a linear model. On the other hand, a mixed model can deal with repeated measurements and treat the missing data as a random loss thus that it will not cause analysis difficulties. If a linear mixed model (LMM) is adopted, it cannot consider the high degree of nonlinearity even if it can deal with repeated measurements and missing value. As a result, this study used the generalized additive mixed model (GAMM) to solve this nonlinear, missing value processing, and individual susceptibility difference analysis of repetitive measurement data [19,20,21,22]. The overall model is given in the form:

y_i_ = **X_i_β** + f_1_(x_1i_) + f_2_(x_2i_) + … + **Z_i_b** + ε_i_, (1)

where y_i_ is the HRV-related parameter, **X_i_** is a fixed-effects design matrix, **β** is a fixed-effects vector, f is the smooth function of the covariate x_k_, **Z_i_** is a random-effects design matrix, **b** is a random-effects vector, and ε is the residual covariance matrix.

In order to assess the impact of the personal pollutant exposures on HRV indices, using the GAMM, this study put PM_1_, PM_1–2.5_, PM_2.5–10_, NP, BC, *p*-PAHs, and CO into multi-pollutant models to carry out the assessment and to control the confounding effect of other pollutants on the target pollutants. With regard to the random effect of the model, the study controlled the parameter of the subjects. In terms of fixed effects, after referring relevant literature, this study listed age, gender, body mass index (BMI), temperature, RH, time, and day of the week as confounders [17,18,23,24,25,26,27,28]. In the current study, no seasonal difference was found in the initial analysis. Thus, the factor of the season was not included in the final model to increase statistical power. The literature review also found that many studies used the hourly moving average concentration of pollutants to observe their “cumulative” health effects, and the shortest one was usually the 1 h moving average concentration [29,30]. Therefore, this study selected 9 time scales (5 min average concentration, 1, 2, 3, 4, 5, 6, 7, and 8 h moving average concentration), and the R statistical software (version 2.10.1, R Foundation for Statistical Computing, Vienna, Austria) was used to model the personal exposures of nine time scales and HRVs with the GAMM. The independent variables were personal pollutant exposures of various moving time scales, and the dependent variable was the natural logarithm of each 5 min interval HRV parameter, including ln HR, ln r-MSSD, ln SDNN, ln pNN50+1, ln LF, ln HF, ln TP, and ln LF/HF. To observe the influence of pollutants on a certain physiological index, the results were expressed with a pollutant coefficient (β) that had been obtained through model analysis using the formula: (e^β^ − 1) × 100% [18].

## 3. Results

### 3.1. Summary Statistics for Personal Monitoring

There were 7 subjects in this study, including 3 males and 4 females, with the age of 68.7 ± 9.0 years (mean ± SD) and a BMI of 23.6 ± 2.5 kg/m^2^. All subjects had no issues with mobility, and their BMI values were within the normal range. Personal exposures, including PM_10_, PM_2.5–10_, PM_2.5_, PM_1–2.5_, PM_1_, NP, *p*-PAHs, BC, CO, temperature, and RH were calculated in 5 min segments. The exposure distribution of the subjects (excluding their sleeping time) is shown in Table 1. The exposure concentrations of PM_10_, PM_2.5_, PM_1_, and NP were 37.72 ± 29.15 µg/m^3^, 29.95 ± 23.47 µg/m^3^, 24.44 ± 19.91 µg/m^3^, and 29,700 ± 46,810 pt/cm^3^, respectively. Personal exposures to particulate components of *p*-PAHs and BC were 13.22 ± 40.69 ng/m^3^ and 2324 ± 2152.54 ng/m^3^, respectively, while the CO exposure concentration was 0.67 ± 0.69 ppm. According to the Taiwan Environmental Protection Agency’s (Taiwan EPA) Indoor Air Quality Standards, the 24 h standard value of PM_10_ is 75 μg/m^3^, the 24 h standard value of PM_2.5_ is 35 μg/m^3^, and the 8 h standard value of CO is 9 ppm [31]. Given this, the mean personal exposures of PM_10_, PM_2.5_, and CO did not exceed the standard values in the current study for administrative purposes. In addition, based on the peak time of each pollutant exposure, this research determined pollution sources by referring to time-activity logs of all subjects. It has found that incense burning and cooking were the main culprits of indoor pollution, where each subject spent 30 min on average on relevant activities every day.

### 3.2. Effects of Personal Pollutant Exposures on HRV Indices

As shown in Table 1, the mean and SD of each HRV parameter were as follows: ln HR of 4.34 ± 0.14 beat/min, ln SDNN of 3.66 ± 0.52 ms, ln r-MSSD of 3.19 ± 0.59 ms, ln pNN50 + 1 of 1.36 ± 0.95%, ln LF of 5.24 ± 1.16 ms^2^, ln HF of 4.44 ± 1.21 ms^2^, ln TP of 6.76 ± 1.10 ms^2^, and ln LF/HF of 0.81 ± 0.86. In addition, the results of the multi-pollutant models could fall into four categories (Table A1, Table A2, Table A3, Table A4, Table A5, Table A6, Table A7 and Table A8). PM_2.5–10_, PM_1_, and NP led to increases in HR, time-domain indices (r-MSSD, SDNN, and pNN50 + 1), and frequency-domain indices (LF, HF, and TP), while causing a decrease in LF/HF. The main HRV effects of PM_1–2.5_ and BC were HR declined, time-domain indices (r-MSSD, SDNN, and pNN50 + 1) declined, frequency-domain indices (LF, HF, and TP) raised, and LF/HF raised. Exposure to *p*-PAHs could lead to HR rise. Finally, the main HRV impacts of CO were HR declined, time-domain indices (r-MSSD, SDNN, and pNN50 + 1) declined, and frequency-domain indices (LF, HF, and TP) declined.

**Figure 1:**
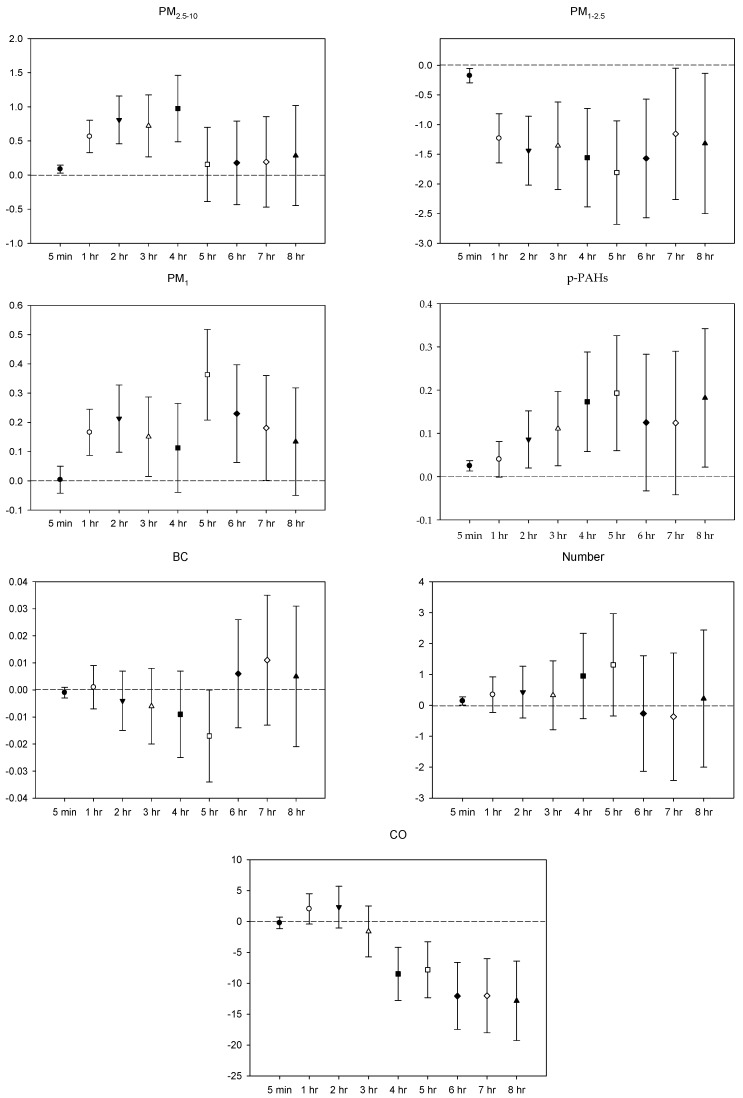
Effects of personal pollutant exposures on HR under the multi-pollutant model. The *X*-axis represents the moving average time of exposure. The *Y*-axis represents the change in HRV percentage for each unit increase in pollutant concentration (BC is the parts per thousand change). The box center represents the mean, and the upper/lower bounds represent 95% confidence interval.

**Figure 2:**
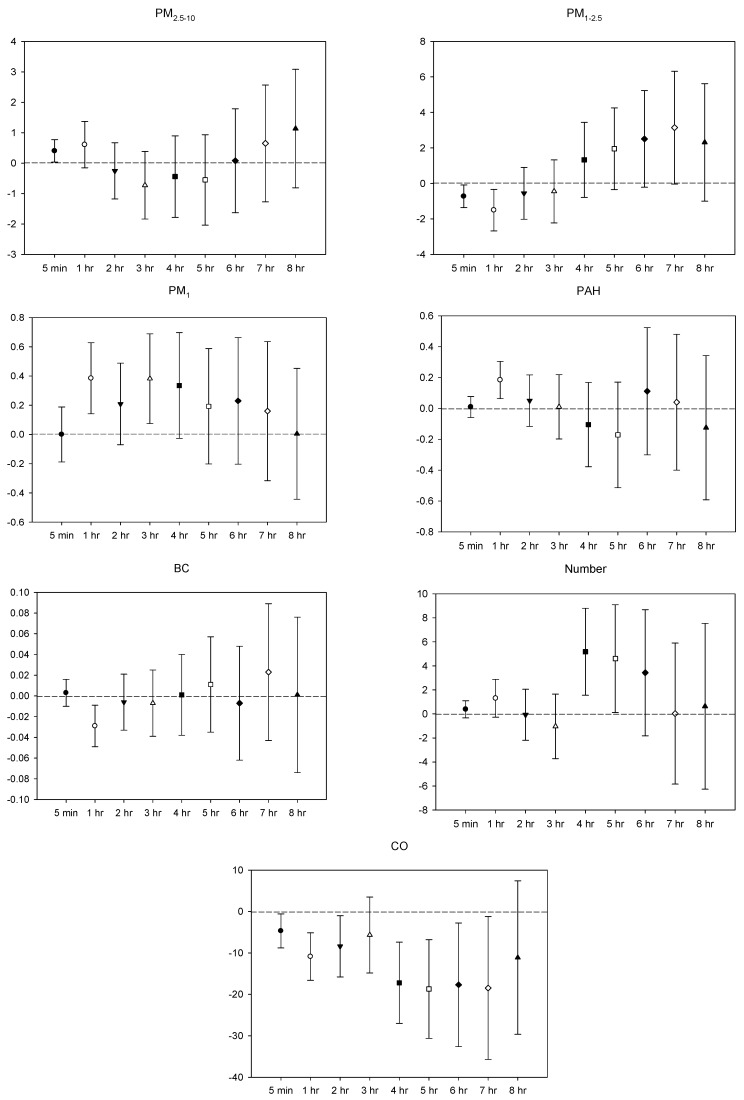
Effects of personal pollutant exposures on SDNN under the multi-pollutant model. The *X*-axis represents the moving average time of exposure. The *Y*-axis represents the change in HRV percentage for each unit increase in pollutant concentration (BC is the parts per thousand change). The box center represents the mean, and the upper/lower bounds represent 95% confidence interval.

**Figure 3:**
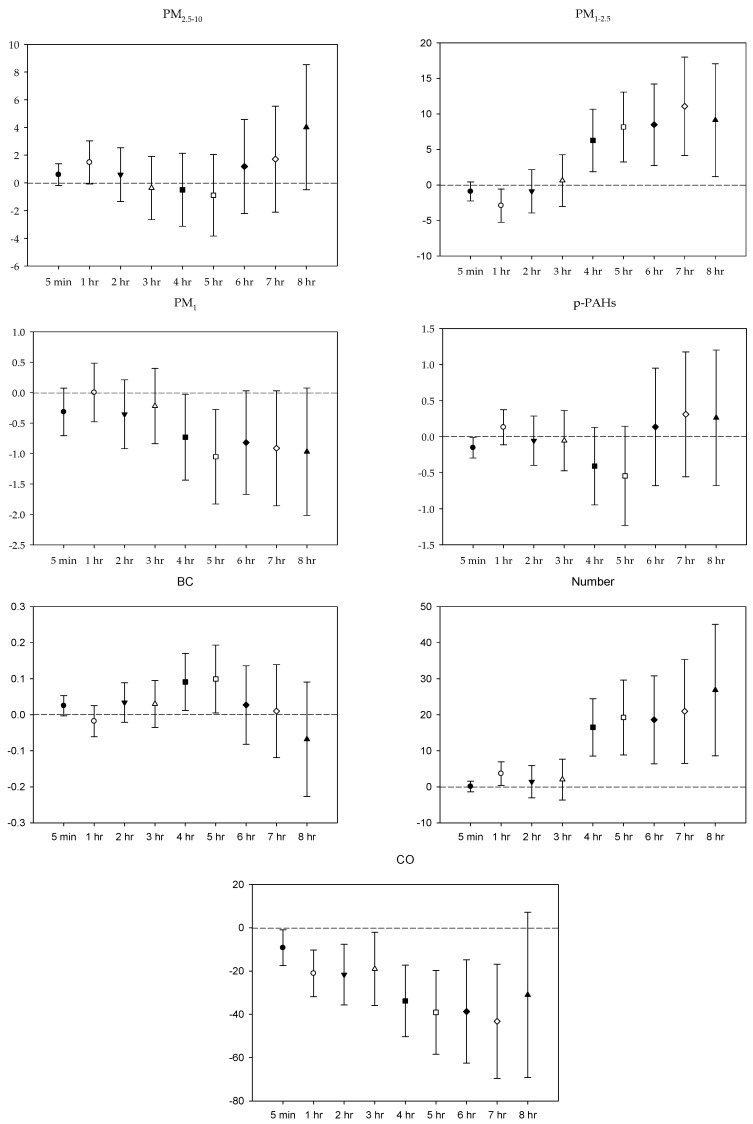
Effects of personal pollutant exposures on LF under the multi-pollutant model. The *X*-axis represents the moving average time of exposure. The *Y*-axis represents the change in HRV percentage for each unit increase in pollutant concentration (BC is the parts per thousand change). The box center represents the mean, and the upper/lower bounds represent 95% confidence interval.

**Figure 4:**
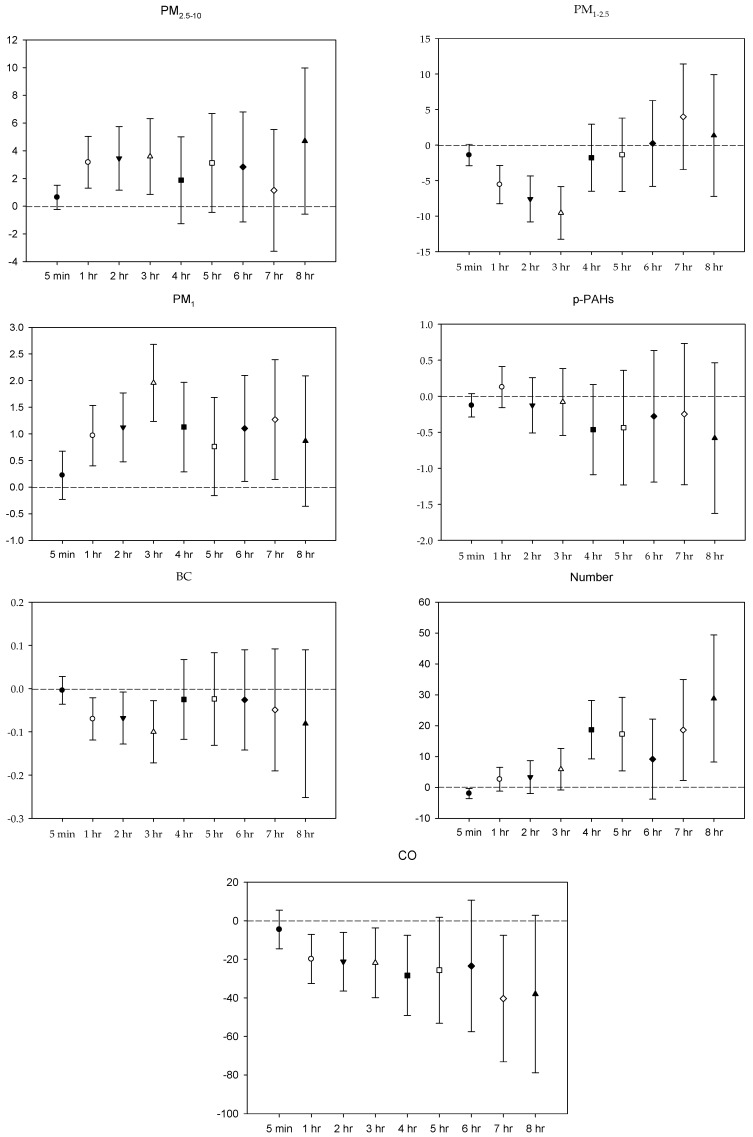
Effects of personal pollutant exposures on HF under the multi-pollutant model. The *X*-axis represents the moving average time of exposure. The *Y*-axis represents the change in HRV percentage for each unit increase in pollutant concentration (BC is the parts per thousand change). The box center represents the mean, and the upper/lower bounds represent 95% confidence interval.

In the multi-pollutant models, the significant increases in HR with the increase in the moving average concentrations were caused by 5 min and 1–4 h exposures to PM_2.5–10_, 1–3 h and 5-7 h PM_1_ exposure, 5 min, 2–5 h, and 8 h exposures to *p*-PAHs, and 5 min NP exposure. Meanwhile, there were 5 min and 1–8 h exposures to PM_1–2.5_, and 4–8 h exposures to CO that led to significant declines in HR as the moving average concentrations rose (Figure 1). As to SDNN, significant increases were caused by 5 min PM_2.5–10_ exposure, 7 h PM_1–2.5_ exposure, 1 h and 3 h PM_1_ exposures, 1 h *p*-PAHs exposure, and 4 h and 5 h exposures to NP. The significant declines in SDNN were caused by 5 min and 1 h exposures to PM_1–2.5_, 5 min, 1–2 h and 4–7 h CO exposures, and 1 h exposure to BC (Figure 2). For LF, the significant increases in LF were caused by 4–8 h exposures to PM_1–2.5_, 1 h and 4–8 h NP exposures, and 4–5 h exposures to BC; its significant declines resulted from 1 h PM_1-2.5_ exposure, 4–5 h exposures to PM_1_, 5 min exposure to *p*-PAHs, and 5 min and 1–7 h exposures to CO (Figure 3). Finally, the significant increases in HF with the increase in the moving average exposures were caused by 1–3 h exposures to PM_2.5–10_, 1–4 h, and 6–7 h exposures to PM_1_, and 4–5 h and 7–8 h exposures to NP. Its declines were related to 1–3 h exposures to PM_1–2.5_, 1–4 h and 7 h exposures to CO, 5-min NP exposure, and 1–3 h exposures to BC (Figure 4).

## 4. Discussion

Compared with previous research results, the pollutant exposure levels in this research were at the same order of magnitude. Chan et al. monitored 9 healthy adults and 10 seniors with pulmonary dysfunction, whose average concentrations of ultrafine particles (20–1000 nm) stood at 23,407 pt/cm^3^ and 25,529 pt/cm^3^, respectively, while the NP exposures in the current study were 29,700 pt/cm^3^ [23]. Jia et al. evaluated 30 older people and found that their median concentration of PM_2.5_ exposure was 44.09 µg/m^3^ compared to 23.07 µg/m^3^ in this research [24]. Schwartz et al. found out that the median concentrations of BC and CO exposures of 28 seniors were 1.2 µg/m^3^ and 0.45 ppm, respectively [25]. The median concentrations of BC and CO exposures in this research were 1.9 µg/m^3^ and 0.53 ppm, respectively; both are slightly higher than the research results of Schwartz et al.

In the current study, based on peak exposure and activity-log records, it was found that incense burning and cooking were the main contributing factors for indoor pollution. Huang et al. reported that indoor PM levels were associated with decreased HRV among housewives. After adjustment for confounders, an interquartile range (IQR) increase in PM_2.5_ was associated with statistically significant 1.25–4.31% decreases in SDNN and 0.12–3.71% decreases in r-MSSD, and these effects were stronger during stir-frying, cleaning with detergent, and burning incense [32]. Results from our study add to the growing evidence that air pollutants from incense burning and cooking can induce autonomic dysfunction in human subjects similar to those from vehicle and industrial emissions, suggesting certain caution in cooking activity and the use of incense for older people [25,27,33]. The public health implication is momentous as high levels of exposure to indoor air pollution from cooking, and incense burning are common in Asian countries [34].

Results of the multi-pollutant models of this study can be compared with the study on HRV effects of 20 healthy young adults’ commuting exposures in Taiwan [22]. The latter indicated that an increase of 1 µg/m^3^ in the moving average concentration of 5 min PM_2.5–10_ exposure would result in a significant increase of 0.055% in HR, while this study had a 0.088% increase, and a significant increase in SDNN at 0.082%, while 0.402% in this study. An increase of 1 µg/m^3^ in the moving average concentration of 1 h PM_2.5–10_ exposure would cause a significant increase of 0.169% in HR versus 0.566% in this study. A 1 µg/m^3^ increase in the moving average concentration of PM_1–2.5_ at 5 min exposure would result in a significant decrease of 0.137% in HR, while 0.175% in this study. The significant decrease in SDNN for 5 min PM_1–2.5_ exposure in the latter was 0.513% versus 0.726% in this study. An increase of 1 µg/m^3^ in the moving average concentration of 1 h PM_1–2.5_ exposure would cause a significant decrease of 0.994% in HR in the latter and 1.230% in this study. An increase of 1 µg/m^3^ in the moving average concentration of 1 h PM_1_ exposure would result in a significant increase of 0.125% in HR in the latter and 0.166% in this study. At last, an increase of 1 ppm in the moving average concentration of 1 h CO exposure would cause a significant decrease of 1.844% in SDNN in the latter, while 10.872% in this study. Accordingly, comparing HRV impacts caused by pollutant exposures of the two studies, it could be seen that whether in terms of PM or CO, the changes in HRV in this study were relatively large with several times the effect difference. It is suggested that the difference is due to the age of the subjects. The ability of cardiac function of the young population to adapt to the external environment is better while the older population is rather susceptible to air pollutants.

Although most studies in the past have focused on the health effects of PM_10_ and PM_2.5_, later studies have started to investigate the effects of finer size particles. Chan et al. measured the effects of finer particles in healthy adults and older patients with pulmonary dysfunction. After controlling the confounding factors such as age, gender, BMI, cigarette exposure, and temperature, it was found that for every increase of 10,000 pt/cm^3^ in the 2 h and 3 h moving average exposures to ultrafine particles (particle size of 0.02–1 µm), the SDNN of older patients with pulmonary dysfunction decreased significantly by 3.00% and 2.87%, and the LF decreased significantly by 5.04% and 4.35%, respectively. The SDNN (down 0.073% and 1.038%) and LF (up 1.403% and 2.027%) changes of healthy older people in this study did not reach statistical significance. The study of Chan et al. also found that every 10,000 pt/cm^3^ increase in the 1–3 h moving average exposures to ultrafine particles would cause a significant HF decrease of 3.61%, 5.61%, and 4.97%, respectively, for older patients with pulmonary dysfunction. Yet the HF changes in this study were not statistically significant (up 2.865%, 3.357%, and 5.910%, respectively) [23]. From the above results, it can be seen that compared with older people with pulmonary dysfunction, their healthy counterparts in this study had a small percentage in HRV change after exposure to NP, indicating that poor health conditions would increase the susceptibility of cardiovascular damage of particulate pollutants.

In addition, the review study by Weichenthal has shown that in observational studies among elderly men and healthy adults, in which exposure was assessed using personal monitoring, there was a suggestion of a negative association between parasympathetic regulation of the heart (i.e., HF, r-MSSD, pNN50) and ultrafine particles, but that the findings from studies of controlled humans exposure suggested no association. Reasons for these discrepancies may include differences in particle composition, time-point of clinical evaluation, and population susceptibilities [35]. In fact, accumulated evidence to date suggests that ultrafine particle and PM_2.5_ can induce acute pathophysiological responses [36]. Kan et al. proposed that pulmonary exposure to engineered nanoparticles may cause detrimental effects on the cardiovascular system by three general mechanisms: Translocation, inflammatory response, and neuronal regulation. Due to their complex nature, engineered nanoparticles may not utilize only one of the proposed mechanisms but act as some combination of the three to bring about changes in the cardiovascular system [37].

This study also found that the moving average concentrations of 1–3 h exposures to PM_1_ would cause a significant increase in the frequency-domain indicator HF, while the moving average concentration of 4–8 h PM_1–2.5_ exposure would result in the rise of the HRV indicators LF and SDNN. These results are also comparable to findings from previous research. A panel study by Wheeler et al. evaluated 18 older patients with chronic lung obstruction aged 49–76 years old. They found that after controlling BMI, temperature, RH, age, gender, drug use, week, and season, an increase of 11.65 µg/m^3^ in the moving average concentration of 4 h PM_2.5_ exposure would result in a significant increase of 35.88% in LF and a significant increase of 8.29% in SDNN [26]. Another panel study by Riediker et al. investigated nine young healthy men aged 23–30 years and found that after controlling CO, nitrogen dioxide (NO_2_), and RH, the 9 h PM_2.5_ exposure was 24.1 µg/m^3^, in which an increase of 10 µg/m^3^ would cause a significant increase in SDNN of 11.7% [33]. In addition, research by Jia et al. studied 30 older people aged between 51 and 73 years and found that with the control of gender, age, BMI, daily time, HR, temperature, RH, and activity types, every 10 µg/m^3^ increase in PM_2.5_ concentration would result in a significant increase of 1.30% in HF and a significant increase of 1.34% in LF [24]. In fact, the cardiovascular effects of PM investigated in animal studies also supported these relative findings. It was speculated that PM_2.5_ deposited in lungs would stimulate the C afferent fiber from bronchi to the lungs and regulate the cardiopulmonary parasympathetic reflex function. Then it would make the parasympathetic stimulation dominant, reflecting an increase in the HRV frequency-domain indices [38]. The increase in HRV indices caused by particulate exposure mainly reflects that the increase of parasympathetic activity has been confirmed to be associated with sinoatrial node dysfunction and vagal response (including potentially fatal arrhythmias and paroxysmal atrial fibrillation) [39].

In terms of HRV effects of CO exposure, the decline of SDNN, r-MSSD, and HF caused by CO in this study was consistent with findings from previous studies. Schwartz et al. studied 27 older people aged 61–89 with no symptoms of unstable angina pectoris, auricular flutter, or atrial fibrillation. It was found that after controlling the drug use status, time, days of the week, temperature, and smoking history, every increase of one IQR (0.45 ppm) in the 24 h average concentration of CO resulted in a significant decrease of 4.2% in SDNN [25]. Timonen et al. evaluated 131 older people with an average age of 68.1 years and found that after controlling temperature, RH, and days of the week, every 1 mg/m^3^ increase in CO exposure concentration resulted in a significant decrease of 5.69% in SDNN and 30.7% in HF [40]. Besides, results of animal studies indicated that CO could change the respiratory rhythm driven by the central nervous system, resulting in slight changes in respiratory regulation, which will then affect HRV [41,42].

After integrating the results, it was found that the average time points of maximum HRV percentage change caused by various pollutants all fell after 1 h and might appear at 8 h. It was speculated that HRV effects were delayed after exposure to size-fractionated PM, *p*-PAHs, BC, and CO. The delayed effects were similar to the study of Magari et al., which investigated the SDNN effect of PM_2.5_ exposure in 40 boiler workers aged 19–59 years and the study of Chang et al. which looked into the HRV effects of PM exposures in 15 older people [27,28]. Magari et al. found that after controlling time, smoking status, and age, the moving average time of PM_2.5_ appeared at 9 h when SDNN showed the maximum significant decline. Every 1000 µg/m^3^ increase in PM_2.5_ would cause a significant decrease in SDNN by 13.01%. When HR showed the maximum significant decline, the moving average time point of PM_2.5_ appears at 7 h. Every 1000 µg/m^3^ increase in PM_2.5_ would cause a significant decrease in HR by 9.3% [27]. Additionally, Chang et al. found that after controlling gender, age, BMI, temperature, RH, time, and disease status, PM_2.5–10_ had the strongest association with the time-domain index (SDNN) and frequency-domain indices (LF, HF) at different moving average time points. The most significant HRV changes in moving average point occurred during the 5–8 h periods. The strongest HRV effect occurred during the moving average time point of PM_2.5–10_ at 6 h. Every 1 µg/m^3^ increase in PM_2.5–10_ caused a significant decrease in SDNN by 1.43% [28].

Overall, this study found that different pollutants caused the rise or fall of HRV indices in different ways, echoing the inconsistent trend found in previous research. It was suggested that the inconsistency might be due to the two different ways of how air pollutants affect the ANS. The first is that the response of the SNS to stress leads to an increase in HR and a decrease in HRV indices, while the second is that the stimulation of the lungs and respiratory receptors leads to the enhancement of the vagal control of the cardiac autonomic rhythm, which will be reflected in the increase in HRV indices [43].

The advantage of this study lies in the short-term continuous repeated measurement data, which can be directly linked with the HRV indices as a representative analysis, and this cannot be achieved with data from fixed monitoring stations. Additionally, the individual continuous monitoring data also represent that each individual case can be used as its own control group to effectively explore the possible effects of multivariate exposure factors on people’s health through statistical analysis [44]. Although there are other studies of this type, the current study is one of the few in Asia, especially for PM of different sizes and their components, thus our work is an important contribution from this perspective [15,16,17,32].

On the other hand, the major limitation of this panel study includes the small number of study participants, which would limit the generalizability of the study to an older population. To maximize statistical power in the small number of subjects, repeated measurements were used, and GAMM methodology was adopted to detect the associations between size-fractioned PM (and its constituents) and HRV. Other possible limitations might also confound our findings, including unavailable data of some key physiologic and environmental information. First, we could not adjust for respiration-modulated autonomic activity in our study because we did not measure key respiration parameters, such as nasal and mouth airflow, chest wall movement, and abdominal movement [32]. Second, our findings could not rule out effects of unmeasured air pollutants, such as ozone (O_3_) and NO_2_, on cardiovascular health [24,45,46,47]. Third, recruitment by posters might result in selection bias. Compared to individuals who choose not to enroll in such studies, those willing to enroll tend to be in better health and good socioeconomic status. Further studies are warranted to assess whether other population groups may respond differently to the same level of exposure. Last, the confounding effects of noise on HRV require further consideration because researchers have pointed out that noise may be one of the factors that potentially interfere with the autonomic nervous system [48].

## 5. Conclusions

In this study, the exposure concentrations of PM_10_, PM_2.5_, and CO during the monitoring period did not exceed the respective standards of indoor air quality. According to the records of time-activity logs, incense burning and cooking were the main indoor pollution sources. Results of multi-pollutant model analyses showed that PM_2.5–10_, PM_1_, and NP all increased HR, the time-domain indices, and the frequency-domain indices; both of PM_1–2.5_ and BC would cause the rise of frequency-domain indices; *p*-PAHs led to an increase in HR; and CO caused the drop in the time-domain and the frequency-domain indices. As for the average time points of maximum HRV percentage change caused by various pollutants, it was found that they all fell after one hour and might appear at 8 h. Exposures to size-fractionated PM, *p*-PAHs, BC, and CO were suggested to have delayed effects on HRV. In addition, the results of this study showed that the increased exposure levels of personal pollutants would affect the cardiac volitional control function in healthy older adults, and their susceptibility to cardiovascular effects was higher than their younger counterparts. Finally, since the present research is limited in the small number of study participants, which would reduce the generalizability of the research findings, future studies are warranted to further investigate the HRV effects of simultaneous PM and other pollution exposures for subpopulation groups with larger scale.

## Figures and Tables

**Table 1:** Summary statistics of heart rate variability indices and personal exposures for the participants.

Variable ^#^	*N*	Mean	Median	SD	Minimum	Maximum
ln HR (beat/min)	4740	4.34	4.35	0.14	3.91	4.83
Time-domain HRV						
ln SDNN (ms)	4740	3.66	3.66	0.52	2.08	7.57
ln r-MSSD (ms)	4740	3.19	3.09	0.59	0.69	7.76
ln pNN50 + 1 (%)	4740	1.36	1.31	0.95	0.00	4.56
Frequency-domain HRV						
ln LF (ms^2^)	4740	5.24	5.28	1.16	1.18	12.37
ln HF (ms^2^)	4740	4.44	4.41	1.21	1.00	12.13
ln TP (ms^2^)	4740	6.76	6.78	1.10	3.17	13.44
ln LF/HF	4740	0.81	0.87	0.86	−2.39	3.61
Personal exposures *						
PM_10_ (µg/m^3^)	4737	37.72	30.85	29.15	2.00	683.00
PM_2.5–10_ (µg/m^3^)	4737	7.77	6.27	9.25	0.00	298.00
PM_2.5_ (µg/m^3^)	4737	29.95	23.07	23.47	1.00	386.00
PM_1–2.5_ (µg/m^3^)	4737	5.51	4.28	6.87	0.00	206.00
PM_1_ (µg/m^3^)	4737	24.44	18.40	19.91	1.00	179.00
NP (pt/cm^3^)	4383	29,700	16,664	46,810	434	586,154
*p*-PAHs (ng/m^3^)	4734	13.22	6.67	40.69	0.00	641.00
CO (ppm)	4624	0.67	0.53	0.69	0.00	11.00
BC (ng/m^3^)	4252	2324.00	1919.67	2152.54	73.00	46,089.00
Temperature (°C)	4738	25.25	26.62	5.21	17.00	37.00
RH (%)	4738	71.83	72.00	9.54	50.00	94.00

^#^ HR: heart rate. HRV: heart rate variability. r-MSSD: square root of the mean of the sum of the squares of differences between adjacent normal-to-normal (NN) intervals. SDNN: standard deviation of all NN intervals. pNN50: percentage of successive NN interval differences greater than 50 ms. LF: low-frequency power. HF: high-frequency power. TP: total power. NP: nanoparticle. p-PAHs: particle-bound polycyclic aromatic hydrocarbons. CO: carbon monoxide. BC: black carbon. RH: relative humidity; * Personal exposures were 5 min averages.

## Appendix A

**Table A1:** Effects of personal pollutant exposures on HR (multi-pollutant model) ^+^.

Interval	PM_2.5–10_	PM_1–2.5_	PM_1_	*p*-PAHs	CO	NP	BC
5 min	0.088 *	−0.175 *	0.004	0.025 *	−0.237	0.142 *	−0.0001
	(0.029, 0.146)	(−0.295, −0.055)	(−0.042, 0.050)	(0.013, 0.037)	(−1.169, 0.704)	(0.009, 0.276)	(−0.0003, 0.0001)
1 h	0.566 *	−1.230 *	0.166 *	0.040	2.034	0.346	0.0001
	(0.328, 0.803)	(−1.644, −0.815)	(0.087, 0.245)	(−0.002, 0.081)	(−0.369, 4.495)	(−0.225, 0.920)	(−0.0006, 0.0009)
2 h	0.808 *	−1.438 *	0.213 *	0.086 *	2.331	0.430	−0.0004
	(0.458, 1.158)	(−2.014, −0.858)	(0.098, 0.328)	(0.021, 0.152)	(−0.949, 5.719)	(−0.401, 1.268)	(−0.0015, 0.0007)
3 h	0.721 *	−1.357 *	0.151 *	0.111 *	−1.627	0.328	−0.0006
	(0.270, 1.174)	(−2.089, −0.619)	(0.015, 0.287)	(0.025, 0.197)	(−5.573, 2.485)	(−0.774, 1.442)	(−0.0020, 0.0008)
4 h	0.976 *	−1.556 *	0.113	0.173 *	−8.484 *	0.952	−0.0009
	(0.491, 1.463)	(−2.379, −0.726)	(−0.038, 0.265)	(0.059, 0.288)	(−12.580, −4.197)	(−0.409, 2.332)	(−0.0025, 0.0007)
5 h	0.155	−1.808 *	0.363 *	0.193 *	−7.821 *	1.313	−0.0017
	(−0.386, 0.698)	(−2.673, −0.936)	(0.209, 0.518)	(0.060, 0.326)	(−12.129, −3.302)	(−0.315, 2.967)	(−0.0034, 0.00002)
6 h	0.180	−1.569 *	0.230 *	0.125	−12.059 *	−0.264	0.0006
	(−0.429, 0.792)	(−2.558, −0.569)	(0.063, 0.397)	(−0.032, 0.283)	(−17.155, −6.649)	(−2.099, 1.606)	(−0.0014, 0.0026)
7 h	0.193	−1.155 *	0.181 *	0.124	−12.023 *	−0.367	0.0011
	(−0.464, 0.855)	(−2.249, −0.049)	(0.002, 0.360)	(−0.041, 0.290)	(−17.631, −6.035)	(−2.389, 1.696)	(−0.0013, 0.0035)
8 h	0.286	−1.317 *	0.134	0.182 *	−12.847 *	0.220	0.0005
	(−0.441, 1.018)	(−2.485, −0.135)	(−0.050, 0.318)	(0.023, 0.342)	(−18.829, −6.424)	(−1.950, 2.438)	(−0.0021, 0.0031)

^+^ Coefficients are expressed as % change (95 confidence interval) in HRV for increase in pollutant exposures (per 1 µg/m^3^ for PM; per 1 ng/m^3^ for *p*-PAHs and BC; per 1 ppm for CO; per 10,000 pt/cm^3^ for NP) in models after adjusting for age, gender, BMI, temperature, RH, time, and day of the week. * *p* < 0.05.

**Table A2:** Effects of personal pollutant exposures on r-MSSD (multi-pollutant model) ^+^.

Interval	PM_2.5–10_	PM_1–2.5_	PM_1_	*p*-PAHs	CO	NP	BC
5 min	0.731 *	−1.605 *	0.193	−0.015	−4.897	−0.042	−0.0009
	(0.287, 1.178)	(−2.384, −0.820)	(−0.048, 0.434)	(−0.098, 0.068)	(−9.629, 0.083)	(−0.915, 0.839)	(−0.0025, 0.0007)
1 h	1.729 *	−3.615 *	0.705 *	0.154	−12.226 *	2.050	−0.0048 *
	(0.722, 2.745)	(−5.102, −2.105)	(0.410, 1.001)	(−0.001, 0.308)	(−18.865, −5.043)	(−0.058, 4.202)	(−0.0073, −0.0022)
2 h	1.606 *	−4.591 *	0.966 *	0.138	−14.521 *	2.615	−0.0052 *
	(0.353, 2.874)	(−6.421, −2.726)	(0.608, 1.326)	(−0.077, 0.353)	(−22.470, −5.757)	(−0.272, 5.585)	(−0.0085, −0.0019)
3 h	1.207	−5.528 *	1.415 *	0.033	−10.846 *	2.329	−0.0052 *
	(−0.196, 2.630)	(−7.536, −3.477)	(1.055, 1.776)	(−0.215, 0.281)	(−19.787, −0.908)	(−1.050, 5.823)	(−0.0087, −0.0017)
4 h	0.519	−2.490	1.387 *	−0.003	−22.601 *	9.774 *	−0.0052 *
	(−1.211, 2.279)	(−5.068, 0.157)	(0.916, 1.859)	(−0.357, 0.352)	(−33.308, −10.176)	(5.030, 14.732)	(−0.0102, −0.0002)
5 h	1.461	−4.180 *	1.483 *	0.118	−23.241 *	6.770 *	−0.0082 *
	(−0.393, 3.348)	(−6.765, −1.524)	(1.030, 1.938)	(−0.301, 0.538)	(−34.943, −9.434)	(1.368, 12.460)	(−0.0133, −0.0030)
6 h	1.478	−4.081 *	1.539 *	0.144	−24.753 *	6.015	−-0.0077 *
	(−0.597, 3.596)	(−7.046, −1.022)	(1.040, 2.039)	(−0.367, 0.658)	(−38.183, −8.407)	(−0.262, 12.687)	(−0.0139, −0.0014)
7 h	1.792	−3.881 *	1.774 *	0.159	−29.911 *	8.662 *	−0.0099 *
	(−0.611, 4.253)	(−7.385, −0.244)	(1.190, 2.360)	(−0.414, 0.736)	(−44.839, −10.943)	(0.882, 17.041)	(−0.0182, −0.0017)
8 h	3.360 *	−5.081 *	1.420 *	−0.162	−32.968 *	18.625 *	−0.0127 *
	(0.604, 6.192)	(−9.075, −0.913)	(0.792, 2.051)	(−0.776, 0.456)	(−48.984, −11.924)	(8.477, 29.723)	(−0.0230, −0.0024)

^+^ Coefficients are expressed as % change (95 confidence interval) in HRV for increase in pollutant exposures (per 1 µg/m^3^ for PM; per 1 ng/m^3^ for *p*-PAHs and BC; per 1 ppm for CO; per 10,000 pt/cm^3^ for NP) in models after adjusting for age, gender, BMI, temperature, RH, time, and day of the week. * *p* < 0.05.

**Table A3:** Effects of personal pollutant exposures on SDNN (multi-pollutant model) ^+^.

Interval	PM_2.5–10_	PM_1–2.5_	PM_1_	*p*-PAHs	CO	NP	BC
5 min	0.402 *	−0.726 *	0.0003	0.009	−4.668 *	0.383	0.0003
	(0.034, 0.770)	(−1.359, −0.089)	(−0.188, 0.189)	(−0.059, 0.077)	(−8.607, −0.559)	(−0.320, 1.090)	(−0.0011, 0.0016)
1 h	0.607	−1.503 *	0.385 *	0.184 *	−10.872 *	1.301	−0.0029 *
	(−0.149, 1.368)	(−2.659, −0.332)	(0.144, 0.628)	(0.064, 0.305)	(−16.245, −5.155)	(−0.250, 2.877)	(−0.0050, −0.0009)
2 h	−0.254	−0.554	0.209	0.050	−8.396 *	−0.073	−0.0006
	(−1.167, 0.668)	(−1.991, 0.905)	(−0.068, 0.488)	(−0.118, 0.217)	(−15.232, −1.008)	(−2.157, 2.054)	(−0.0033, 0.0021)
3 h	−0.725	−0.449	0.382 *	0.010	−5.663	−1.038	−0.0007
	(−1.823, 0.386)	(−2.190, 1.323)	(0.076, 0.689)	(−0.197, 0.218)	(−14.024, 3.511)	(−3.652, 1.648)	(−0.0039, 0.0025)
4 h	−0.441	1.330	0.335	−0.105	−17.197 *	5.175 *	0.0001
	(−1.763, 0.900)	(−0.744, 3.447)	(−0.026, 0.697)	(−0.378, 0.168)	(−25.962, −7.393)	(1.677, 8.794)	(−0.0038, 0.0040)
5 h	−0.551	1.954	0.193	−0.172	−18.698 *	4.607 *	0.0011
	(−2.016, 0.935)	(−0.295, 4.253)	(−0.199, 0.588)	(−0.514, 0.170)	(−29.075, −6.802)	(0.304, 9.094)	(−0.0034, 0.0057)
6 h	0.079	2.507	0.230	0.111	−17.684 *	3.428	−0.0007
	(−1.599, 1.785)	(−0.144, 5.229)	(−0.201, 0.663)	(−0.299, 0.523)	(−30.300, −2.784)	(−1.566, 8.676)	(−0.0061, 0.0048)
7 h	0.649	3.141 *	0.160	0.040	−18.470 *	0.030	0.0023
	(−1.232, 2.567)	(0.059, 6.319)	(−0.315, 0.636)	(−0.398, 0.480)	(−32.693, −1.241)	(−5.512, 5.897)	(−0.0042, 0.0089)
8 h	1.136	2.315	0.005	−0.125	−11.122	0.639	0.0001
	(−0.773, 3.083)	(−0.890, 5.625)	(−0.441, 0.453)	(−0.590, 0.342)	(−26.447, 7.397)	(−5.818, 7.538)	(−0.0075, 0.0076)

^+^ Coefficients are expressed as % change (95 confidence interval) in HRV for increase in pollutant exposures (per 1 µg/m^3^ for PM; per 1 ng/m^3^ for *p*-PAHs and BC; per 1 ppm for CO; per 10,000 pt/cm^3^ for NP) in models after adjusting for age, gender, BMI, temperature, RH, time, and day of the week. * *p* < 0.05.

**Table A4:** Effects of personal pollutant exposures on pNN50 + 1 (multi-pollutant model) ^+^.

Interval	PM_2.5–10_	PM_1–2.5_	PM_1_	*p*-PAHs	CO	NP	BC
5 min	0.738 *	−2.032 *	0.476 *	−0.082	−0.481	−0.360	−0.0011
	(0.090, 1.389)	(−3.224, −0.826)	(0.074, 0.880)	(−0.208, 0.045)	(−8.498, 8.238)	(−1.708, 1.007)	(−0.0035, 0.0014)
1 h	2.872 *	−5.759 *	1.125 *	0.105	−17.221 *	5.325 *	−0.0076 *
	(1.088, 4.689)	(−8.365, -3.078)	(0.577, 1.677)	(−0.175, 0.386)	(−28.547, −4.100)	(1.449, 9.349)	(−0.0124, −0.0028)
2 h	2.302 *	−6.767 *	1.266 *	0.125	−22.159 *	6.474 *	−0.0075 *
	(0.073, 4.581)	(−9.946, −3.476)	(0.601, 1.935)	(−0.263, 0.515)	(−35.086, −6.657)	(1.198, 12.025)	(−0.0136, −0.0013)
3 h	2.526	−8.287 *	1.972 *	0.183	−18.560	4.813	−0.0085 *
	(−0.127, 5.251)	(−11.942, −4.481)	(1.243, 2.707)	(−0.288, 0.656)	(−34.174, 0.758)	(−1.563, 11.602)	(−0.0157, −0.0013)
4 h	0.604	−4.732 *	1.984 *	−0.042	−28.709 *	10.628 *	−0.0063
	(−2.374, 3.672)	(−9.124, −0.128)	(1.147, 2.829)	(−0.669, 0.590)	(−45.101, −7.423)	(2.330, 19.598)	(−0.0151, 0.0025)
5 h	2.302	−7.276 *	2.105 *	−0.263	−23.593	5.520	−0.0069
	(−1.143, 5.868)	(−11.979, −2.321)	(1.183, 3.035)	(−1.066, 0.546)	(−44.684, 5.539)	(−4.321, 16.373)	(−0.0175, 0.0036)
6 h	2.201	−7.158 *	1.984 *	−0.634	−21.361	4.362	−0.0032
	(−1.639, 6.191)	(−12.509, -1.479)	(0.988, 2.991)	(−1.575, 0.315)	(−46.066, 14.660)	(−6.857, 16.934)	(−0.0155, 0.0092)
7 h	3.214	−9.688 *	2.664 *	−0.710	−25.629	7.271	−0.0090
	(−0.943, 7.544)	(−15.396, −3.595)	(1.633, 3.706)	(−1.698, 0.288)	(−50.761, 12.331)	(−5.731, 22.066)	(−0.0233, 0.0054)
8 h	7.200 *	−12.934 *	2.753 *	−0.640	−21.792	13.394	−0.0129
	(2.335, 12.298)	(−19.113, −6.282)	(1.654, 3.864)	(−1.671, 0.401)	(−50.571, 23.743)	(−2.420, 31.771)	(−0.0303, 0.0046)

^+^ Coefficients are expressed as % change (95 confidence interval) in HRV for increase in pollutant exposures (per 1 µg/m^3^ for PM; per 1 ng/m^3^ for *p*-PAHs and BC; per 1 ppm for CO; per 10,000 pt/cm^3^ for NP) in models after adjusting for age, gender, BMI, temperature, RH, time, and day of the week. * *p* < 0.05.

**Table A5:** Effects of personal pollutant exposures on LF (multi-pollutant model) ^+^.

Interval	PM_2.5–10_	PM_1–2.5_	PM_1_	*p*-PAHs	CO	NP	BC
5 min	0.595	−0.912	−0.316	−0.153 *	−9.241 *	0.099	0.0025
	(−0.182, 1.378)	(−2.230, 0.424)	(−0.706, 0.075)	(−0.296, −0.010)	(−16.808, −0.985)	(−1.358, 1.579)	(−0.0003, 0.0053)
1 h	1.481	−2.902 *	0.004	0.130	−21.085 *	3.660 *	−0.0018
	(−0.050, 3.036)	(−5.187, −0.562)	(−0.475, 0.485)	(−0.112, 0.373)	(−30.585, −10.285)	(0.482, 6.940)	(−0.0061, 0.0025)
2 h	0.592	−0.878	−0.354	−0.056	−21.667 *	1.403	0.0034
	(−1.308, 2.529)	(−3.834, 2.170)	(−0.916, 0.212)	(−0.397, 0.286)	(−33.558, −7.648)	(−2.865, 5.858)	(−0.0022, 0.0089)
3 h	−0.367	0.632	−0.219	−0.055	−19.023 *	2.027	0.0030
	(−2.595, 1.912)	(−2.873, 4.264)	(−0.833, 0.399)	(−0.472, 0.363)	(−33.012, -2.112)	(−3.345, 7.699)	(−0.0035, 0.0095)
4h	−0.499	6.276 *	−0.731 *	−0.409	−33.847 *	16.521 *	0.0091 *
	(−3.063, 2.133)	(2.049, 10.679)	(−1.430, −0.026)	(−0.944, 0.129)	(−47.062, −17.333)	(9.075, 24.475)	(0.0012, 0.0170)
5 h	−0.893	8.163 *	−1.051 *	−0.545	−39.128 *	19.244 *	0.0099 *
	(−3.752, 2.050)	(3.460, 13.080)	(−1.823, −0.274)	(−1.229, 0.144)	(−53.817, −19.767)	(9.707, 29.610)	(0.0005, 0.0193)
6 h	1.186	8.482 *	−0.820	0.135	−38.713 *	18.602 *	0.0027
	(−2.094, 4.575)	(3.035, 14.216)	(−1.663, 0.031)	(−0.674, 0.950)	(−55.899, −14.830)	(7.545, 30.795)	(−0.0081, 0.0136)
7 h	1.710	11.082 *	−0.913	0.310	−43.255 *	20.925 *	0.0010
	(−1.973, 5.532)	(4.571, 17.999)	(−1.848, 0.031)	(−0.549, 1.176)	(−61.287, −16.823)	(8.051, 35.332)	(−0.0119, 0.0139)
8 h	4.020	9.130 *	−0.969	0.262	−31.037	26.846 *	−0.0068
	(−0.302, 8.529)	(1.725, 17.074)	(−2.002, 0.076)	(−0.669, 1.202)	(−55.630, 7.186)	(10.905, 45.078)	(−0.0228, 0.0091)

^+^ Coefficients are expressed as % change (95 confidence interval) in HRV for increase in pollutant exposures (per 1 µg/m^3^ for PM; per 1 ng/m^3^ for *p*-PAHs and BC; per 1 ppm for CO; per 10,000 pt/cm^3^ for NP) in models after adjusting for age, gender, BMI, temperature, RH, time, and day of the week. * *p* < 0.05.

**Table A6:** Effects of personal pollutant exposures on HF (multi-pollutant model) ^+^.

Interval	PM_2.5–10_	PM_1–2.5_	PM_1_	*p*-PAHs	CO	NP	BC
5 min	0.638	−1.403	0.224	−0.128	−4.498	−1.930 *	−0.0004
	(−0.231, 1.515)	(−2.891, 0.107)	(−0.225, 0.676)	(−0.289, 0.034)	(−13.564, 5.519)	(−3.552, −0.281)	(−0.0035, 0.0028)
1 h	3.169 *	−5.556 *	0.969 *	0.126	−19.828 *	2.658	−0.0070 *
	(1.334, 5.038)	(−8.170, −2.867)	(0.405, 1.536)	(−0.158, 0.411)	(−30.844, −7.058)	(−1.064, 6.519)	(−0.0119, −0.0021)
2 h	3.455 *	−7.585 *	1.123 *	−0.127	−21.195 *	3.357	−0.0068 *
	(1.207, 5.754)	(−10.706, −4.354)	(0.481, 1.769)	(−0.510, 0.258)	(−33.938, −5.995)	(−1.693, 8.667)	(−0.0128, −0.0008)
3 h	3.592 *	−9.543 *	1.958 *	−0.080	−21.769 *	5.910	−0.0100 *
	(0.930, 6.324)	(−13.101, −5.840)	(1.240, 2.680)	(−0.540, 0.383)	(−36.470, −3.666)	(−0.402, 12.622)	(−0.0171, −0.0028)
4 h	1.880	−1.779	1.129 *	−0.464	−28.337 *	18.697 *	−0.0025
	(−1.162, 5.017)	(−6.289, 2.948)	(0.295, 1.970)	(−1.085, 0.161)	(−44.489, −7.486)	(9.940, 28.151)	(−0.0117, 0.0067)
5 h	3.122	−1.358	0.763	−0.437	−25.651	17.281 *	−0.0024
	(−0.330, 6.693)	(−6.262, 3.803)	(−0.149, 1.683)	(−1.227, 0.358)	(−45.739, 1.875)	(6.483, 29.174)	(−0.0132, 0.0083)
6 h	2.832	0.235	1.105 *	−0.280	−23.433	9.179	−0.0026
	(−0.993, 6.805)	(−5.453, 6.266)	(0.120, 2.100)	(−1.183, 0.632)	(−47.042, 10.702)	(−2.400, 22.132)	(−0.0143, 0.0090)
7 h	1.138	3.981	1.269 *	−0.249	−40.345 *	18.602 *	−0.0049
	(−3.069, 5.529)	(−2.955, 11.413)	(0.158, 2.392)	(−1.219, 0.731)	(−61.506, −7.553)	(4.267, 34.906)	(−0.0189, 0.0092)
8 h	4.705	1.350	0.867	−0.581	−38.010	28.801 *	−0.0081
	(−0.317, 9.981)	(−6.540, 9.906)	(−0.343, 2.091)	(−1.616, 0.464)	(−62.629, 2.826)	(11.056, 49.382)	(−0.0251, 0.0090)

^+^ Coefficients are expressed as % change (95 confidence interval) in HRV for increase in pollutant exposures (per 1 µg/m^3^ for PM; per 1 ng/m^3^ for *p*-PAHs and BC; per 1 ppm for CO; per 10,000 pt/cm^3^ for NP) in models after adjusting for age, gender, BMI, temperature, RH, time, and day of the week. * *p* < 0.05.

**Table A7:** Effects of personal pollutant exposures on LF/HF (multi-pollutant model) ^+^.

Interval	PM_2.5–10_	PM_1–2.5_	PM_1_	*p*-PAHs	CO	NP	BC
5 min	−0.172	0.611	−0.613 *	−0.009	−5.283	2.055 *	0.0027 *
	(−0.844, 0.505)	(−0.538, 1.774)	(−0.940, −0.286)	(−0.130, 0.114)	(−12.019, 1.969)	(0.781, 3.344)	(0.0003, 0.0051)
1 h	−1.350 *	2.436 *	−0.966 *	0.094	−1.374	0.770	0.0035
	(−2.599, −0.084)	(0.418, 4.494)	(−1.363, −0.568)	(−0.109, 0.297)	(−11.327, 9.695)	(−1.825, 3.433)	(−0.0001, 0.0071)
2 h	−2.215 *	6.412 *	−1.536 *	0.141	1.559	−2.129	0.0078 *
	(−3.737, −0.669)	(3.818, 9.071)	(−1.993, −1.077)	(−0.138, 0.422)	(−10.862, 15.711)	(−5.529, 1.393)	(0.0032, 0.0124)
3 h	−2.064 *	9.158 *	−2.114 *	0.105	3.043	−2.734	0.0111 *
	(−3.848, −0.248)	(6.046, 12.362)	(−2.606, −1.621)	(−0.236, 0.448)	(−11.421, 19.869)	(−6.963, 1.687)	(0.0060, 0.0163)
4 h	−1.546	8.551 *	−2.157 *	0.026	1.057	−3.180	0.0119 *
	(−3.629, 0.582)	(4.965, 12.260)	(−2.721, −1.590)	(−0.424, 0.479)	(−15.923, 21.465)	(−8.439, 2.380)	(0.0057, 0.0182)
5 h	−2.568 *	9.714 *	−2.218 *	−0.120	−7.076	−0.654	0.0133 *
	(−4.837, −0.246)	(5.826, 13.746)	(−2.830, −1.602)	(−0.688, 0.452)	(−25.858, 16.463)	(−7.293, 6.461)	(0.0060, 0.0207)
6 h	−0.809	8.714 *	−2.285 *	0.366	−10.837	5.611	0.0057
	(−3.312, 1.759)	(4.389, 13.218)	(−2.949, −1.617)	(−0.291, 1.027)	(−31.135, 15.445)	(−2.378, 14.253)	(−0.0026, 0.0140)
7 h	0.847	8.211 *	−2.408 *	0.570	1.952	3.488	0.0017
	(−1.803, 3.570)	(3.463, 13.176)	(−3.092, −1.718)	(−0.100, 1.244)	(−22.665, 34.405)	(−5.008, 12.745)	(−0.0078, 0.0112)
8 h	0.876	7.056 *	−1.693 *	1.029 *	5.004	−1.321	−0.0022
	(−1.891, 3.721)	(1.968, 12.397)	(−2.348, −1.034)	(0.312, 1.752)	(−19.880, 37.618)	(−10.647, 8.978)	(−0.0135, 0.0092)

^+^ Coefficients are expressed as % change (95 confidence interval) in HRV for increase in pollutant exposures (per 1 µg/m^3^ for PM; per 1 ng/m^3^ for *p*-PAHs and BC; per 1 ppm for CO; per 10,000 pt/cm^3^ for NP) in models after adjusting for age, gender, BMI, temperature, RH, time, and day of the week. * *p* < 0.05.

**Table A8:** Effects of personal pollutant exposures on total power (TP) (multi-pollutant model) ^+^.

Interval	PM_2.5–10_	PM_1–2.5_	PM_1_	*p*-PAHs	CO	NP	BC
5 min	0.849 *	−1.555 *	0.188	0.017	−10.210 *	0.274	−0.0004
	(0.094, 1.610)	(−2.818, −0.275)	(−0.192, 0.570)	(−0.122, 0.156)	(−17.328, −2.479)	(−1.137, 1.705)	(−0.0031, 0.0023)
1 h	1.598 *	−3.391 *	0.540 *	0.291 *	−21.290 *	3.413 *	−0.0039
	(0.129, 3.087)	(−5.561, −1.171)	(0.071, 1.011)	(0.059, 0.523)	(−30.158, −11.296)	(0.397, 6.519)	(−0.0079, 0.0002)
2 h	0.373	−1.652	0.124	0.091	−21.219 *	0.798	0.0007
	(−1.398, 2.176)	(−4.382, 1.155)	(−0.414, 0.664)	(−0.233, 0.416)	(−32.210, −8.446)	(−3.223, 4.986)	(−0.0045, 0.0059)
3 h	0.169	−2.439	0.529	0.071	−22.190 *	1.907	−0.0010
	(−1.939, 2.322)	(−5.606, 0.835)	(−0.063, 1.125)	(−0.314, 0.457)	(−34.391, −7.720)	(−3.160, 7.239)	(−0.0069, 0.0049)
4 h	0.179	2.330	0.036	−0.435	−35.969 *	13.088 *	0.0061
	(−2.275, 2.693)	(−1.517, 6.327)	(−0.633, 0.708)	(−0.934, 0.067)	(−47.848, −21.383)	(6.199, 20.425)	(−0.0010, 0.0131)
5 h	−0.483	4.319 *	−0.183	−0.495	−40.238 *	13.315 *	0.0071
	(−3.199, 2.310)	(0.075, 8.742)	(−0.914, 0.553)	(−1.124, 0.139)	(−53.589, −23.046)	(4.750, 22.580)	(−0.0011, 0.0154)
6 h	0.390	6.020 *	−0.146	0.048	−41.222 *	12.064 *	0.0047
	(−2.683, 3.560)	(1.046, 11.240)	(−0.942, 0.655)	(−0.688, 0.790)	(−56.468, −20.636)	(2.276, 22.789)	(−0.0048, 0.0142)
7 h	1.747	6.793 *	−0.212	0.082	−48.232 *	10.816	0.0072
	(−1.765, 5.384)	(0.884, 13.048)	(−1.107, 0.692)	(−0.713, 0.884)	(−63.743, −26.086)	(−0.186, 23.031)	(−0.0042, 0.0187)
8 h	3.388	6.677	−0.162	0.260	−42.137 *	16.393 *	0.0009
	(−0.740, 7.688)	(−0.221, 14.053)	(−1.150, 0.837)	(−0.608, 1.134)	(−61.608, −12.792)	(3.066, 31.442)	(−0.0131, 0.0150)

^+^ Coefficients are expressed as % change (95 confidence interval) in HRV for increase in pollutant exposures (per 1 µg/m^3^ for PM; per 1 ng/m^3^ for *p*-PAHs and BC; per 1 ppm for CO; per 10,000 pt/cm^3^ for NP) in models after adjusting for age, gender, BMI, temperature, RH, time, and day of the week. * *p* < 0.05.
